# NIPU: a randomised, open-label, phase II study evaluating nivolumab and ipilimumab combined with UV1 vaccination as second line treatment in patients with malignant mesothelioma

**DOI:** 10.1186/s12967-021-02905-3

**Published:** 2021-05-31

**Authors:** Vilde Drageset Haakensen, Anna K. Nowak, Espen Basmo Ellingsen, Saima Jamil Farooqi, Maria Moksnes Bjaanæs, Henrik Horndalsveen, Tine Mcculloch, Oscar Grundberg, Susana M. Cedres, Åslaug Helland

**Affiliations:** 1 Department of Oncology, Oslo University Hospital, Oslo, Norway; 2 Department of Cancer Genetics, Institute for Cancer Research, Oslo University Hospital, Oslo, Norway; 3 National Centre for Asbestos Related Diseases, Institute for Respiratory Health, University of Western Australia, Perth, Australia; 4 Department of Medical Oncology, Sir Charles Gairdner Hospital, Nedlands, Australia; 5 Department of Tumor Biology, Institute for Cancer Research, Oslo University Hospital, Oslo, Norway; 6 Ultimovacs, Oslo, Norway; 7 Department of Oncology and Clinical Cancer Research Center, Aalborg University Hospital, Aalborg, Denmark; 8 Department of Clinical Medicine, Faculty of Medicine, Aalborg University, Aalborg, Denmark; 9 Thoracic Oncology Center, Theme Cancer, Karolinska University Hospital, Stockholm, Sweden; 10 Oncology Department, Vall d’Hebron University Hospital and Vall d’Hebron Institute of Oncology, Barcelona, Spain; 11 Faculty of Medicine, University of Oslo, Oslo, Norway

**Keywords:** Malignant pleural mesothelioma, Telomerase vaccine, Immunotherapy, hTERT, Nivolumab, Ipilimumab, Biomarker, Immune response

## Abstract

**Background:**

Malignant pleural mesothelioma (MPM) is a rare and aggressive tumour. For patients with inoperable disease, few treatment options are available after first line chemotherapy. The combination of ipilimumab and nivolumab has recently shown increased survival compared to standard chemotherapy, but most patients do not respond and improvements are called for. Telomerase is expressed in mesothelioma cells, but only sparsely in normal tissues and is therefore an attractive target for therapeutic vaccination. Vaccination against telomerase is tolerable and has shown to induce immune responses associated with increased survival in other cancer types. There is a well-founded scientific rationale for the combination of a telomerase vaccine and checkpoint inhibition to improve treatment response in MPM patients.

**Methods:**

NIPU is a randomized, multi-centre, open-label, phase II study comparing the efficacy and safety of nivolumab and ipilimumab with or without telomerase vaccine in patients with inoperable malignant pleural mesothelioma after first-line platinum-based chemotherapy. Participants (n = 118) are randomized 1:1 into two treatment arms. All participants receive treatment with nivolumab (240 mg every 2 weeks) and ipilimumab (1 mg/kg every 6 weeks) until disease progression, unacceptable toxicity or for a maximum of 2 years. Patients randomised to the experimental arm receive 8 intradermal injections of UV1 vaccine during the first three months of treatment. Tumour tissue, blood, urine, faeces and imaging will be collected for biomarker analyses and exploration of mechanisms for response and resistance to therapy.

**Discussion:**

Checkpoint inhibition is used for treatment of mesothelioma, but many patients still do not respond. Increasing therapy response to immunotherapy is an important goal. Possible approaches include combination with chemotherapy, radiotherapy, targeted therapy and other immunotherapeutic agents. Predictive biomarkers are necessary to ensure optimal treatment for each patient and to prevent unnecessary side effects. This trial seeks to improve treatment response by combining checkpoint inhibition with a telomerase vaccine and also to explore mechanisms for treatment response and resistance. Knowledge gained in the NIPU study may be transferred to the first line setting and to other cancers with limited benefit from immunotherapy.

*Trial registration*: ClinicalTrials.gov: NCT04300244, registered March 8th, 2020, https://clinicaltrials.gov/ct2/show/NCT04300244?term=NIPU&draw=2&rank=1.

## Background

Malignant pleural mesothelioma (MPM) is a rare and aggressive tumour originating from the cells lining the mesothelial surface in the lungs. Median overall survival (OS) is ~ 1 year [1]. Asbestos exposure is linked to development of the disease [2]. Most patients diagnosed with malignant pleural mesothelioma are inoperable and have been treated with cisplatin in combination with pemetrexed which has been the standard of care first-line therapy worldwide since 2003.

Intra-tumour infiltration of CD8+ T cells is associated with improved outcome in MPM [3] and a high expression of PD-L1 is associated with poor prognosis in this group of patients [4]. Monotherapy using pembrolizumab, nivolumab or avelumab has demonstrated a response rate of 9.3–20% [5–7]. The only phase 3 trial combining PD-1 and CTLA4 inhibition (Checkmate 743) showed an increased median survival from 14.1 to 18.1 months, and a duration of response of 11.0 vs 6.7 months in the combination arm vs the chemotherapy arm, respectively [8]. However, almost all the benefit was seen in patients with biphasic or sarcomatoid disease, whereas those with epithelioid disease did not benefit significantly. Grade 3 or 4 toxicity with the combination has been found in 17–34% of patients and has generally been manageable. The most common toxicities have been from the skin, gastrointestinal tract, hormone system and infusion reactions [8]. Based on these results, the FDA approved the combination of ipilimumab and nivolumab in the first line setting in October 2020.

Although these results are encouraging, the response rates seen are moderate compared to what has been documented for the combination of checkpoint inhibitors in other cancer diseases, indicating that checkpoint inhibitors alone are not enough to trigger an activation of an immune response against MPM. MPM is often found to be immunologically ‘cold’ tumours (lacking immune cell infiltration), but previous studies have indicated that treatment can increase immune cell infiltration in such cold MPM tumours by oncolytic virus [9]. Combining checkpoint inhibition with a treatment that can increase lymphocyte infiltration in the tumour could increase the response rates. One such approach is to use a vaccine aiming to activate an immune response directed against tumour-related antigens, and to combine the vaccine with checkpoint inhibitors.

UV1 is a therapeutic cancer vaccine directed against telomerase produced by Ultimovacs ASA. Telomerase maintains telomere length in dividing cells and is considered essential for tumour growth. It is expressed at high levels in more than 85% of human tumours, but only sparsely in normal tissues [10]. Since telomerase is an essential enzyme and universally expressed by most tumour cells, it represents a unique cancer antigen as a basis for immunotherapy.

In the NIPU-trial, UV1 will be tested in combination with ipilimumab and nivolumab to assess its ability to increase the treatment response in patients with MPM in the second line setting. This study was designed prior to the positive results of the CheckMate 743 trial, and may also inform the potential for UV1 vaccination in the first line setting.

## Methods/design

Oslo University Hospital is sponsor of the study. We have established collaborations for patient recruitment with cancer centres in Australia, Denmark, Sweden and Spain. University of Western Australia is sponsor for the study in Australia. Patient recruitment started in June 2020 and is expected to finish by May 2022. Initiation of the trial was postponed due to the COVID-19 pandemic. By March 2021, patients are included in Norway, Australia and Denmark.

### Objectives

#### Primary objective

To evaluate and compare the efficacy of nivolumab and ipilimumab with or without UV1-vaccine in patients with inoperable malignant pleural mesothelioma progressing after first-line platinum-based chemotherapy. The primary end point will be progression free survival (PFS) evaluated by modified response evaluation criteria in solid tumours (mRECIST) [11, 12] as determined by blinded, independent central review (BICR).

#### Secondary objectives

- To compare overall survival (OS), objective response rate (ORR), disease control rate (DCR), time to response (TTR) and duration of response (DOR) according to Response Evaluation Criteria in Solid Tumours, version 1.1 (Modified RECIST), in patients who receive nivolumab and ipilimumab with patients who receive nivolumab and ipilimumab in combination with UV1.
- To evaluate changes from baseline in patient-reported outcomes (PROs) of lung cancer symptoms, patient functioning, and health-related quality of life (HRQoL) in patients who receive nivolumab and ipilimumab compared to patients who receive nivolumab and ipilimumab in combination with UV1.
- To determine tolerability in patients who receive nivolumab and ipilimumab compared to patients who receive nivolumab and ipilimumab in combination with UV1.

#### Exploratory/translational objectives

- To characterise the TCR repertoire in patients receiving checkpoint inhibition alone compared with in combination with UV1 vaccination.
- To evaluate tumour mutational burden (TMB) as a predictive biomarker of response to therapy.
- To assess any difference in immune cell infiltration in tumour pre- and post-treatment with checkpoint inhibition alone or with UV1 vaccination.
- To assess vaccine-specific T-cell response in the blood cells of patients treated with UV1 vaccination.
- To assess any correlation between the microbial composition in faeces and treatment response.
- Evaluate dual-time PET as a predictive marker of therapy response and assess the ability of dual-time PET to distinguish between different cell types in the tumour.

### Study design

This is a randomized, multi-centre, open-label, proof of concept study comparing the efficacy and safety of nivolumab and ipilimumab with or without UV1 in patients with inoperable malignant pleural mesothelioma after first-line platinum-based chemotherapy.

**Fig. 1:**
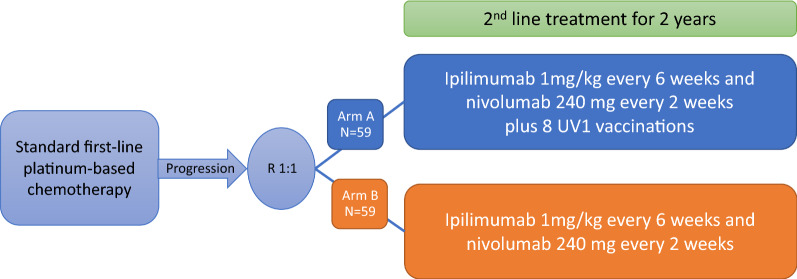
Study design of the NIPU trial. Patients with MPM and progression after first line platinum-based chemotherapy are randomised (1:1) to ipilimumab and nivolumab alone or in combination with UV1 vaccine.

The patients (n = 118) will be randomized 1:1 into two treatment arms (see Fig. 1):

A Nivolumab and ipilimumab plus 8 UV1 vaccinations.
B Nivolumab and ipilimumab alone.

Treatment will be continued until disease progression, unacceptable toxicity or for a maximum of 2 years. Because of the potential for pseudo progression/tumour immune infiltration, this study will allow patients to remain on study treatment after apparent radiographic progression, provided the risk/benefit ratio is judged to be favourable.

Biological material will be sampled, and translational explorative analyses will be performed to identify biomarkers and reveal mechanisms of response and resistance

#### Dosage

- Nivolumab: 240 mg every 2 weeks for 2 years until progression or unacceptable toxicity.
- Ipilimumab: 1 mg/kg every 6 weeks for 2 years until progression or unacceptable toxicity.
- UV1 vaccination (arm A only): 8 intradermal injections with 300 μg UV1 and 75 μg GM-CSF (sargramostim) during the first 13 weeks. The first 3 UV1 vaccinations will begiven in week 1, one in week 2, followed by four UV1 vaccinations throughout the following 11 weeks, totalling eight vaccinations. The first three vaccinations of UV1 will be given prior to the first infusion of nivolumab and ipilimumab.

Overview of the study procedures and treatment can be found in Table 1.

**Table 1:** Overview of study treatment and procedures

Weeks	Study period									
							Up to 2 years	EOT	Q3M safety FU	Q3M survival FU
	–4–0	1	2	3–5	6–7	12	Q6W			
Enrolment										
Informed consent	×									
Randomisation	×									
Treatment arm A										
UV1		× × ×	×	× ×	×	×				
Nivolumab Q2W			×	×	×	× × ×	× × ×			
Ipilimumb Q6W			×		×	×	×			
Treatment arm B										
Nivolumab Q2W		×		× ×	×	× × ×	× × ×			
Ipilimumb Q6W		×			×	×	×			
Procedures										
CT	×				×	×	×	×	×	
Dual-time PET	×				×			×		
Biopsy	×				×			×		
Study blood samples	×				×			×	×	
HR-QoL	×				×	×	×	×		
Clinical e×amination Q2W	×		×	×	×	× × ×	× × ×	×		
AE/SAE Q2W	×		×	×	×	× × ×	× × ×	×		
Disease status/survival									×	×

### Rationale for the choice of vaccine

Telomerase activation is a major factor contributing to cancer proliferation, immortality, and invasiveness [13–15], and its activation is documented in MPM [16, 17]. Tumour telomerase expression is correlated with poor outcome for many cancers [18–20], conversely, spontaneously occurring telomerase-directed CD4+ T-cell responses have recently been identified as a positive prognostic factor in NSLCL [21], substantiating its relevance as a target for vaccination in thoracic malignancies. Vaccination with UV1 induces immune responses directed at telomerase reverse transcriptase (hTERT), the catalytic subunit of the telomerase complex. By targeting this essential cancer protein, there are limited possibilities for resistance mutations to develop, as loss of hTERT would restrict tumour proliferation and metastasis. Furthermore, by mounting a CD4+ Th1 response (i.e. secretion of IFN-γ, TNFα and IL-2), the vaccine aims to induce an inflammatory tumour microenvironment to further stimulate the expansion of secondary effector cells. UV1 is administered with granulocyte-macrophage colony-stimulating factor (GM-CSF) as an adjuvant. The safety and immunological response of the vaccine given as monotherapy have been investigated in two clinical phase I/II trials [22, 23]. The combination of the UV1 vaccine with ipilimumab in metastatic malignant melanoma patients has been investigated in a phase I/IIa trial (n = 12) (Aamdal E. et al. Manus in prep.) (NCT02275416). Together, these studies have shown that UV1 is generally safe and well tolerated. Common adverse events related to UV1 include pruritus, erythema, fatigue, diarrhoea, pain and rash. Overall, six of the 52 patients vaccinated with UV1 (12%) experienced serious adverse events that were related to UV1 and/or GM-CSF, of these five were of allergic origin. All events resolved without sequelae.

Across the three phase I/II-studies conducted with UV1, vaccine-specific immune responses were observed in 78% (range 67–91%) of patients across the different cancer types (and HLA allele types), supporting the universality of the vaccine. Survival time for patients who responded immunologically was longer than for patients who did not respond immunologically to the vaccine, and survival time correlated with the breadth of the vaccine-specific immune response.

In summary, UV1 is safe alone and in combination with checkpoint inhibitors and trials in other cancers show that UV1 induces vaccine-specific immune response associated with survival.

### Rationale for the choice of immunotherapy

The rationale for combining UV1 vaccination with immune checkpoint inhibitors is for the vaccine to prime a meaningful T cell response that can be enhanced by the checkpoint inhibitors to facilitate the activity of the tumour-specific T cells in the tumour microenvironment. Several pre-clinical studies have shown improved anti-tumour responses upon combination of vaccine and checkpoint inhibitors [24]. Data from clinical studies combining anti-immune checkpoint blockade and vaccines are still limited, but some early trials show encouraging results and were recently reviewed [25]. PD-1 and CTLA-4 checkpoints differentially affect CD8+ and CD4+ T-cell phenotypes [26] and recent data indicate that immune responses induced by anti-CTLA-4 and anti-PD-1 checkpoint-blockade are driven by different cellular mechanisms [27]. This indicates that vaccination with long peptides containing both CD4 and CD8 T-cell epitopes combined with both ipilimumab and nivolumab could be expected to have a maximal synergistic effect.

UV1-specific immune responses were induced in 11 out of 12 melanoma-patients treated with UV1 vaccine in combination with ipilimumab. The immune response appeared more frequently and rapidly than in patients treated with UV1 alone [22], [23] (Aamdal et al. manuscript submitted), suggesting a synergistic effect of blocking of CTLA-4 and vaccination with UV1. Of 12 patients, one patient developed a complete response, three patients obtained a partial response, and two achieved stable disease as best overall response. The safety, clinical and immunological responses of the UV1 vaccine when used in combination with the PD-1 immune checkpoint inhibitor pembrolizumab is currently under investigation in a phase I/II clinical trial in patients (N = 30) with metastatic malignant melanoma (NCT03538314).

In summary, ipilimumab and nivolumab has shown clinical benefit in patients with MPM and trials in other tumour groups have found immunological synergy between ipilimumab and UV1.

### Patient selection

Selected inclusion and exclusion criteria are shown in Table 2.

**Table 2:** Selected inclusion and exclusion criteria

Selected inclusion criteria	Selected exclusion criteria
1. Histologically and/or cytologically confirmed MPM	1. Disease suitable for curative surgery
2. Unresectable disease, not candidate for curative surgery	2. Previous treatment with a PD1 or PD-L1 inhibitor
3. Measurable disease, defined as at least one lesion (CT or MRI) that is suitable for repeated assessment	3. Non-pleural mesothelioma e.g. mesothelioma arising in other organs
4. Available unstained archived tumour tissue sample	4. Active second malignancy other than non-melanoma skin cancer or cervical carcinoma in situ
5. Previously treated with at least one line of platinum doublet	5. Symptomatic or uncontrolled brain metastases requiring concurrent treatment
6. ECOG performance status of 0–1	6. Known history of leptomeningeal carcinomatosis
7. Willing to provide archived tumour tissue and blood samples for research	7. Active or prior documented autoimmune or inflammatory disorders
8. Adequate organ function	8. History of primary immunodeficiency
9. Age ≥ 18 years	9. History of allogeneic organ transplant
	10. Uncontrolled intercurrent illness
	11. Active infection

### Safety measures

#### Handling of toxicities

Administration of study treatment will be performed in a setting with emergency medical facilities and staff who are trained to monitor for and respond to medical emergencies. All adverse events and serious adverse events will be recorded during the trial and for up to 30 days after the last dose of study drug or until the initiation of another anti-cancer therapy, whichever occurs first. After this period, investigators should report serious adverse events and adverse events of special interest that are believed to be related to prior treatment with study drug. Events will be graded according to NCI CTCAE v5.0.

#### Dose reduction considerations (selected points)

- Dose reduction of nivolumab or ipilimumab is not permitted, apart from changing the dosing intervals for ipilimumab to every 12th week.
- For any concomitant conditions already apparent at baseline, the dose modifications will apply according to the corresponding shift in toxicity grade, if the investigator feels it is appropriate. For example, if a patient has grade 1 asthenia at baseline that increases to grade 2 during treatment, this will be considered a shift of one grade and treated as grade 1 toxicity for dose-modification purposes.
- When several toxicities with different grades of severity occur at the same time, the dose modifications should be according to the highest grade observed.
- If, in the opinion of the investigator, a toxicity is considered to be due to some component of the treatment leading to a dose delay, the other component(s) may be administered if there is no contraindication.
- If a patient experiences a grade 3 or 4 adverse event considered probably related to ipilimumab, the ipilimumab dosing interval should be extended to every 12 weeks. The ipilimumab dose should be kept at 1mg/kg.
- Patients may temporarily suspend study treatment with nivolumab/ipilimumab if they experience an adverse event that requires a dose to be held.
  - If nivolumab is held because of adverse events for > 42 days (6 weeks), the patient will be discontinued from nivolumab treatment.
  - If ipilimumab is held because of adverse events for > 126 days (18 weeks), the patient will be discontinued from nivolumab treatment.
  - If the patient is likely to benefit from resuming nivolumab/ipilimumab after a longer hold than allowed (6 or 18 weeks), the study drug may be restarted with the approval of the Sponsor. If a patient must be tapered off steroids used to treat adverse events, nivolumab may be held for > 42 days until steroids are discontinued or reduced to prednisone dose equivalent ≤ 10 mg/day.
- The treating physician may use discretion in modifying or accelerating the dose modification guidelines described depending on the severity of toxicity and an assessment of the risk versus benefit for the patient, with the goal of maximizing patient compliance and access to supportive care.
- Dose modification of GM-CSF (sargramostim) or UV1 is not allowed in this study. If ipilimumab and nivolumab are withheld during the study treatment period, UV1 vaccination must also be withheld. UV1 vaccinations will only be reintroduced if both ipilimumab and nivolumab are reintroduced.
- UV1 vaccination and/or ipilimumab and nivolumab not administered will not be replaced during the study treatment period.

#### Independent data monitoring

An independent data monitoring committee (IDMC) has pre-planned assessment of the safety of the trial 4 and 13 weeks after the first 6 and 14 patients have started treatment. All the IDMC assessments has been successfully completed.

### Sample collection/biobanking

An extensive research program will be conducted. The patient informed consent form will allow for performing biomarker analyses, immunological studies, gene profiling and studies of tumour evolution/heterogeneity during treatment, as well as comparison with data/material from other studies.

1. Tumour biopsies collected pre, during and post therapy (time of progression). If sufficient tissue is available, three biopsies will be obtained at each timepoint, and prioritized in the following order:
  - FFPE tissue.
  - Snap-frozen tumour biopsies.
  - Fresh tumour cells/tumour infiltrating lymphocytes frozen as cell suspension (selected sites).
2. A separate biopsy (snap-frozen) will be taken at week 6 from those patients who consent to the procedures.
3. Blood samples collected pre-, during and post-therapy:
  - Peripheral blood mononuclear cells, processed with gradient centrifugation and frozen on liquid nitrogen (selected sites).
  - Plasma/serum, separated and frozen.
  - Circulating tumour cells (only if sufficient resources available).
4. Urine samples collected pre-, during and post-therapy.
5. Faecal samples collected at baseline, tumour evaluation timepoints and progression.
6. Dual-time PET analyses at baseline, 6 weeks, 1 year and at progression (selected sites).

### Statistical analyses

Under the null hypothesis, the PFS hazard ratio (HR), for ipilimumab and nivolumab in combination with UV1 (ipi/nivo/UV1) vs ipilimumab and nivolumab (ipi/nivo) is assumed to be 1.00. Under the alternative hypothesis, the HR is assumed to be 0.60. To test the null hypothesis with 80% power and a 1-sided alpha level of 0.10, a total of 69 PFS events are required. Based on the INITIATE trial [28], with 12 months median follow-up it is expected that 69% of patients treated with ipi/nivo will have progressed and, with a HR of 0.60, it is further expected that 51% of patients treated with ipi/nivo/UV1 will have progressed. With an expected accrual rate of 5 patients per month, a total N=118 patients randomized into the trial over a 24-month period and followed for a minimum of 2–3 months after the last patient is randomized will yield the 69 PFS events required.

## Discussion

The NIPU study is designed as a randomized phase 2 trial. Randomization will provide useful data on both toxicity and efficacy. Since the combination of UV1 with ipilimumab and nivolumab has not previously been studied in patients with MPM, a phase 2 study giving indications of both toxicity and efficacy is warranted before starting a phase 3 trial for both ethical and economic reasons. The trial is open-labelled as placebo vaccination with observation time was impractical and time-consuming for both patients and nurses and blinding was not considered necessary for a phase 2 trial with a blinded independent centrally reviewed primary endpoint.

As treatment guidelines change, the patient population eligible for clinical trials change. One challenge with the NIPU trial is that patients treated with checkpoint inhibition in first line are not eligible for the trial and once the ipilimumab/nivolumab treatment regime is implemented, the patient population eligible for the study will rapidly reduce. Until that time, however, the patients have few treatment options in the second line and the clinical data gained from this trail may be relevant for the first line setting in the future. Inclusion has started and shows that patients are eager to participate in the trial.

The translational analyses constitute an important part of the trial. As immunotherapy is being established as part of the treatment available to patients with MPM, it is important to gain knowledge about biological mechanisms contributing to treatment response or resistance.

Tumour biopsies will be collected at baseline and at 6 weeks after start of treatment to evaluate the tumour microenvironment and its association with therapy effect. Characterization of immune cell subsets will be done in the tumour and in the circulation, including T-cells, B cells, NK cells, myeloid-derived suppressor cells, or subpopulations Immune cells in the tumour will be characterized. Specific immune response against the UV1 vaccine will be analysed as will the changes in clonality of the T cell receptor repertoire in response to therapy.

Previous studies have shown that the temporal dynamics of uptake of 18F-Fluorodeoxyglucose (FDG) varies between benign and malignant lesions [29, 30]. The uptake kinetics of FDG (reflected by glucose consumption rates) may vary between different cell types, and we hypothesize that we can differentiate between cancer and immune cells by so-called dual-time PET [29]. We test this by comparing the cellular composition (tumour cells and various immune cells) at baseline and at 6 weeks after treatment with dual-time PET (scans obtained 60 and 120 min after injection of FDG).

The composition of the gut microbiome has been found associated with response to immunotherapy and the use of antibiotics has been associated with reduced effect of check point inhibition as a result of unfavourable alterations to the gut microbiome [31, 32]. We collect faeces at baseline, 6 weeks, at regular intervals and at progression for DNA extraction and deep sequencing. Microbiota profiles will be linked to immune responses in circulation and in the tumour to identify microbes associated with immune response to treatment. Information about concomitant medication will be available.

Exploration of biological mechanisms of action and biomarker analyses will be performed on tumour tissue, but we will also search for reliable biomarkers using less invasive methods such as blood, imaging, urine and faeces. Based on results from the CheckMate 743 trial indicating more pronounced benefit for sarcomatoid/non-epithelioid tumours [8], the clinical effect and the biological basis for the effect in the epithelioid and non-epithelioid tumours will be explored. The knowledge gained from these translational studies will be of value in understanding the mechanisms for therapy effect and resistance also in the first line setting.

## Data Availability

Not applicable.

## Abbreviations

BICR: Blinded, independent central review
CTCAE: Common terminology criteria for adverse events
CTLA4: Cytotoxic T-lymphocyte-associated protein 4
DCR: Disease control rate
DOR: Duration of response
FDA: Food and drug administration
FFPE: Formalin-fixed paraffin-embedded
GM-CSF: Granulocyte-macrophage colony-stimulating factor
HR: Hazard ratio
HRQoL: Health-related quality of life
MPM: Malignant pleural mesothelioma
mRECIST: Modified response evaluation criteria in solid tumours
NCI: National cancer institute
NIPU: Nivolumab and Ipilimumab +/- UV1 Vaccination as Second Line Treatment in Patients With Malignant Mesothelioma
ORR: Objective response rate
OS: Overall survival
PET: Positron emission tomography
PD-L1: Programmed death-ligand 1
PFS: Progression free survival
PROs: Patient-reported outcomes
TMB: Tumour mutational burden
TTR: Time to response
